# Effects of *Panax quinquefolius* (American ginseng) on the steady state visually evoked potential during cognitive performance

**DOI:** 10.1002/hup.2756

**Published:** 2020-09-08

**Authors:** David J. White, David A. Camfield, Anastasia Ossoukhova, Karen Savage, Romain Le Cozannet, Pascale Fança‐Berthon, Andrew Scholey

**Affiliations:** ^1^ Centre for Human Psychopharmacology Swinburne University Melbourne Victoria Australia; ^2^ Naturex Part of Givaudan Avignon France

**Keywords:** American ginseng, *Panax quinquefolius*, steady state visually evoked potential (SSVEP), working memory

## Abstract

**Objective:**

To investigate the effects of acute *Panax quinquefolius* (American ginseng) administration on steady state visually evoked potentials (SSVEPs) during completion of working memory and continuous performance tasks.

**Methods:**

A randomised, double‐blind, placebo controlled, balanced, cross‐over trial was conducted in middle‐aged volunteers aged between 40 and 60 years. Participants were administered 200 mg *P. quinquefolius* and placebo on two separate testing sessions. Six‐h post‐dose participants completed spatial working memory (SWM) and continuous performance (CP) tasks while SSVEP from a diffuse task‐irrelevant 13 Hz flicker was recorded.

**Results:**

During SWM retrieval, *P. quinquefolius* was associated with significantly reduced prefrontal SSVEP latency. There were no significant treatment effects on CP nor behavioural performance of either task.

**Conclusions:**

These findings provide preliminary evidence of increased recruitment of prefrontal brain regions during working memory processing following a single acute dose of *P. quinquefolius*.

## 1 INTRODUCTION

The longstanding reputation of ginseng as a modulator of cognitive performance is supported by clinical trials. These benefits are thought to arise from the action of ginsenosides, which represent the primary bioactive constituents of ginseng species. Differing ginsenoside profiles across *Panax* species may differentially affect physiological and neurocognitive processes (Smith, Williamson, Putnam, Farrimond, & Whalley, 2014). For example, the ginsenoside Rb_1_ is a cholinergic modulator which is more highly expressed in *Panax quinquefolius* (American ginseng) than the more extensively researched *Panax* species (Asian ginseng).

Most ginseng research in the cognitive arena has focused on *Panax ginseng* (Kennedy et al., 2003; Reay, Kennedy, & Scholey, 2005; Reay, Kennedy, & Scholey, 2006; Reay, Scholey, & Kennedy, 2010; Scholey & Kennedy, 2002). However, two randomised controlled trials (RCTs) have reported enhanced working memory following acute administration of *P. quinquefolius* to healthy cohorts. In younger adults, acute doses of 100, 200 and 400 mg of a standardised *P. quinquefolius* extract (cereboost™) facilitated aspects of cognitive performance (Scholey et al., 2010). Relative to placebo, significant improvements to working memory performance were observed from 1 to 6 h post‐dose. While specific cognitive tasks were differentially enhanced by different doses, spatial working memory (SWM; Corsi blocks) was significantly improved for all doses and all post‐dose timepoints. This led us to evaluate the effects of 200 mg of the same extract in healthy, middle‐aged adults. We again observed enhanced working memory in the 6 h following administration (Ossoukhova et al., 2015). A composite working memory battery was significantly improved and maximal 3 h post‐dose. Inspection of individual tasks revealed, again, SWMas a primary driver of this effect observed in the composite score, albeit on a different task. It should be noted that Ossoukhova et al. (2015) did not observe positive effects on the Corsi blocks task.

In the latter study, neurocognitive function was co‐monitored using steady state visually evoked potentials (SSVEPs) in a subset of participants. Fluctuations in SSVEP amplitude and phase induced by a diffuse visual flicker during cognitive processing have been used to study functional brain activity associated with a range of cognitive processes (Ellis, Silberstein, & Nathan, 2006; Kemp, Gray, Eide, Silberstein, & Nathan, 2002; Silberstein, Ciorciari, & Pipingas, 1995), in addition to their modulation by pharmacological and nutritional intervention (Camfield et al., 2012; White et al., 2016, 2017). Of particular relevance to the present study, reduced SSVEP amplitude and phase have been reported in prefrontal electrode sites during spatial SWM task performance in healthy adults from midlife to older age (Macpherson et al., 2014). Their study reported that this pattern of reduced SSVEP amplitude and latency during SWM performance also indexes a compensatory process in older adults such that greater reductions were associated with better task performance. Here, we report the outcomes of SSVEP assessment in a randomised, double‐blind, placebo controlled, balanced, cross‐over trial of *P. quinquefolius* during completion of this same SWM task reported in Macpherson et al. (2014), in addition to a separate continuous performance (CP) task. Given the previously reported improvements to working memory, we hypothesised that *P. quinquefolius* treatment may modulate activation including the frontal regions during working memory.

## 2 METHODS

### 2.1 Participants

Twenty volunteers (7 Female), aged 40–60 years participated in the SSVEP component of the trial as part of a larger study into the cognitive effects of *P. quinquefolius*, details of which have been reported elsewhere (Ossoukhova et al., 2015) with inclusion and exclusion criteria. Sample size calculations were based off the primary cognitive outcome in the larger clinical trial. The sample size of the SSVEP cohort was chosen to be in line with recent crossover trials investigating the acute neurocognitive effects of nutritional and nutraceutical interventions (Camfield et al., 2012; White et al., 2017). The trial was registered (ACTRN12610000849099), received ethical clearance from the Swinburne University of Technology Human Research Ethics Committee and was conducted according to the Declaration of Helsinki.

### 2.2 Procedure

A randomised, double‐blind, placebo‐controlled, cross‐over acute study measured the cognitive effects of a commercial extract of *P. quinquefolius*. Across two identical counterbalanced testing days, participants were administered 200 mg of *P. quinquefolius* Cereboost^TM^ and an inert placebo. The *P. quinquefolius* treatment contained a standardised 10%–12% ginsenosides administered in the form of an opaque capsule with maltodextrose excipient, while the placebo treatment consisted of an inert plant cellulose fibre (Avicel) that was encapsulated to be identical in appearance (see Scholey et al. (2010) and Supplementary Information (SI‐1) for further details of treatments). The treatments were prepared by a disinterested third party who took no further part in the study. Treatment order was determined by random allocation to a Latin square to ensure a fully counterbalanced design. Cognitive testing with SSVEP recording was conducted 6 h post‐administration (treatment and placebo), following completion of behavioural assessments performed as part of the broader study (Ossoukhova et al., 2015).

### 2.3 Cognitive tasks

Two cognitive tasks were completed during SSVEP recording, an AX‐CP task (AX‐CPT) and a SWM, see Supporting Information SI‐2 for details. These two tasks have been utilised in a series of recent trials investigating modulation of SSVEP responses through pharmaceutical (Silberstein, Pipingas, Farrow, Levy, & Stough, 2016) and nutraceutical interventions (Camfield et al., 2012; White et al., 2016, 2017), with the SWM task probing the specific cognitive domain previously impacted by *P. quinquefolius* (Scholey et al., 2010). Acquisition and pre‐processing of the SSVEP were performed according to White et al. (2016; 2017). Further details of data recording, processing and analyses are provided in Supporting Information SI‐3.

Performance on cognitive tasks was measured as mean response times in correct trials for the active variant of both cognitive tasks and also accuracy of SWM performance. To investigate potential treatment‐related changes in task accuracy and response times, repeated measures analysis of variance (ANOVA), examining the main effect of treatment as a within‐subjects factor, was conducted using SPSS for Windows (Version 23; SPSS Inc). Criteria for significance across behavioural outcomes were set to *p* < .05.

### 2.4 SSVEP analysis

Analyses of SSVEP differences were conducted with Hotelling's T^2^, the bivariate analogue to a paired *T*‐test, in order to simultaneously examine SSVEP amplitude and phase. Components of the active variant of both AX‐CPT and SWM tasks were contrasted between the *P. quinquefolius* treatment visit and the placebo visit, in order to quantify transient changes in SSVEP response during task processing associated with the acute dose of *P. quinquefolius*. Adjustment for multiple comparisons followed previous research to use this SSVEP method, in setting the alpha level for SSVEP analysis to 1% (adjusted *p* = .05/5), based on spatial principal component analysis of SSVEP data (Silberstein & Cadusch, 1992). The association between any significant SSVEP changes and behavioural performance changes from placebo to active treatment was also explored through correlations, in order to explore any behavioural correlates of SSVEP changes.

Additional analyses, characterising SSVEP response to task completion at the placebo visit, contrasting active and reference variants of each task, are detailed in Supporting Information (SI‐5). Furthermore, exploratory analyses to investigate a more tonic shift in SSVEP response associated with treatment were conducted, following methods described in a previous acute nutritional intervention study (White et al., 2017), details and outcomes of this analysis are provided in Supporting Information (SI‐6).

The analysis population for each outcome required complete cases, also excluding datasets with excessive artifact (as defined by objective criteria described in Supporting Information, SI‐4) or task performance approximating chance levels (<55% accuracy). Based on these criteria, the analysis population for the A‐X CPT was *n* = 15 (seven completing treatment sequence A–B and eight B–A) and for the SWM task was *n* = 12 (balanced for treatment order). The exclusions for each task are summarised in Supporting Information (SI‐4).

## 3 RESULTS

Details of the study sample are provided in Table 1. Average behavioural performance for the A‐X CPT and SWM tasks are presented in Table 2. No behavioural performance measure differed statistically between treatments.

**TABLE 1:** Demographic details of the study sample (*n* = 20, 7 female/13 male)

	Mean (±SD)
Age (years)	53.9 ± 5.46
BMI	24.9 ± 2.50
Fasting glucose (mmol/L)	4.97 ± 0.96
Years education	16.40 ± 3.10
MMSE score	28.90 ± 0.76
DASS score	27.60 ± 4.81
STAI‐trait score	32.9 ± 9.47

Abbreviations: BMI, body mass index; MMSE, Mini Mental State Examination; DASS, Depression, Anxiety Stress Scales.

**TABLE 2:** Task performance at placebo and *Panax quinquefolius* treatment visits with results of repeated measures ANOVA

Outcome	Treatment	*M*	SD	*F*	*p*
AX‐CPT‐ response time (ms)	Placebo	396.20	84.76	0.53	.478
	*P. quinquefolius*	385.64	66.75		
SWM—accuracy (%)	Placebo	74.77	5.79	0.30	.596
	*P. quinquefolius*	75.83	4.04		
SWM—response time (ms)	Placebo	848.60	190.19	1.68	.222
	*P. quinquefolius*	809.53	158.51		

*Note: F* and *p* values reported for main effect of treatment visit within repeated measures ANOVA (degrees of freedom for ANOVA: AX‐CPT *F*[1,14]; SWM *F*[1,11]).

Abbreviations: ANOVA, analysis of variance; CPT, continuous process task; SWM, spatial working memory.

**FIGURE 1:**
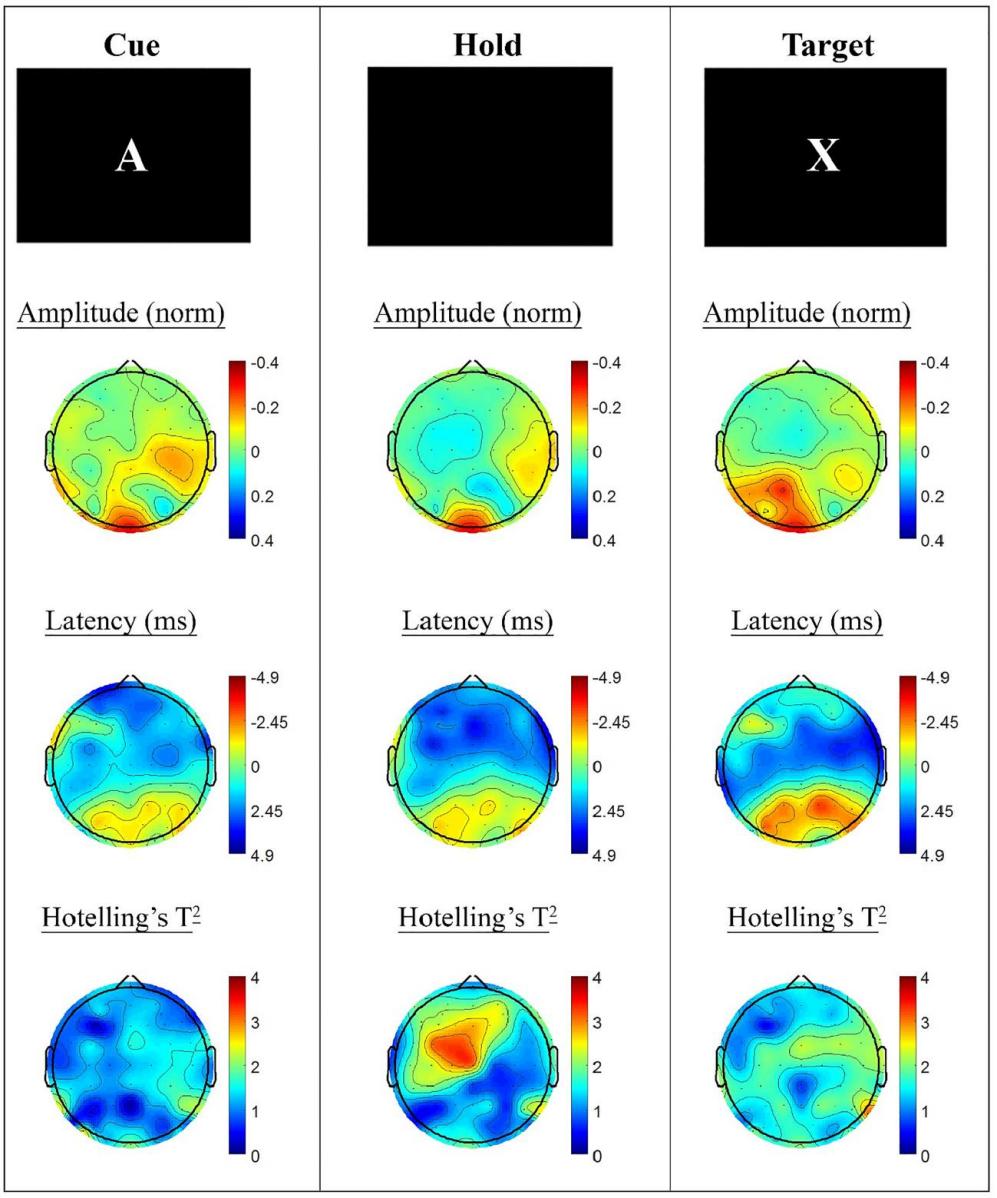
A‐X CPT steady state visually evoked potential (SSVEP) amplitude and phase differences during the active task condition between *Panax quinquefolius* and placebo treatment visits. Columns show the three task blocks, with topographic maps of SSVEP amplitude (top) and phase (as latency in ms, middle) and the Hotelling's T^2^ (bottom) corresponding to the contrast of active and placebo visits for cue (left), hold (middle) and target (right) task segments. Differences are plotted such that negative amplitude and latency values reflect reductions at the active visit. None of these differences reached criteria for statistical significance, with only the fronto–central latency increases during the hold period between cue and target stimuli significant at an uncorrected threshold (*p* < .05; in electrode sites FC3, FCz, C3 and Cz)

Task windows of the active AX‐CPT task were contrasted between the *P. quinquefolius* treatment visit and the placebo visit, in order to quantify transient changes in SSVEP response during task processing associated with the acute dose of *P. quinquefolius*. The overall trend in these comparisons was for amplitude and latency reductions in posterior sites, and increased latency in more anterior regions for the active treatment (see Figure 1). None of these differences reached criteria for statistical significance, with only the fronto–central latency increases during the hold period between cue and target stimuli significant at an uncorrected threshold (*p* <.05; in electrode sites FC3, FCz, C3 and Cz).

**FIGURE 2:**
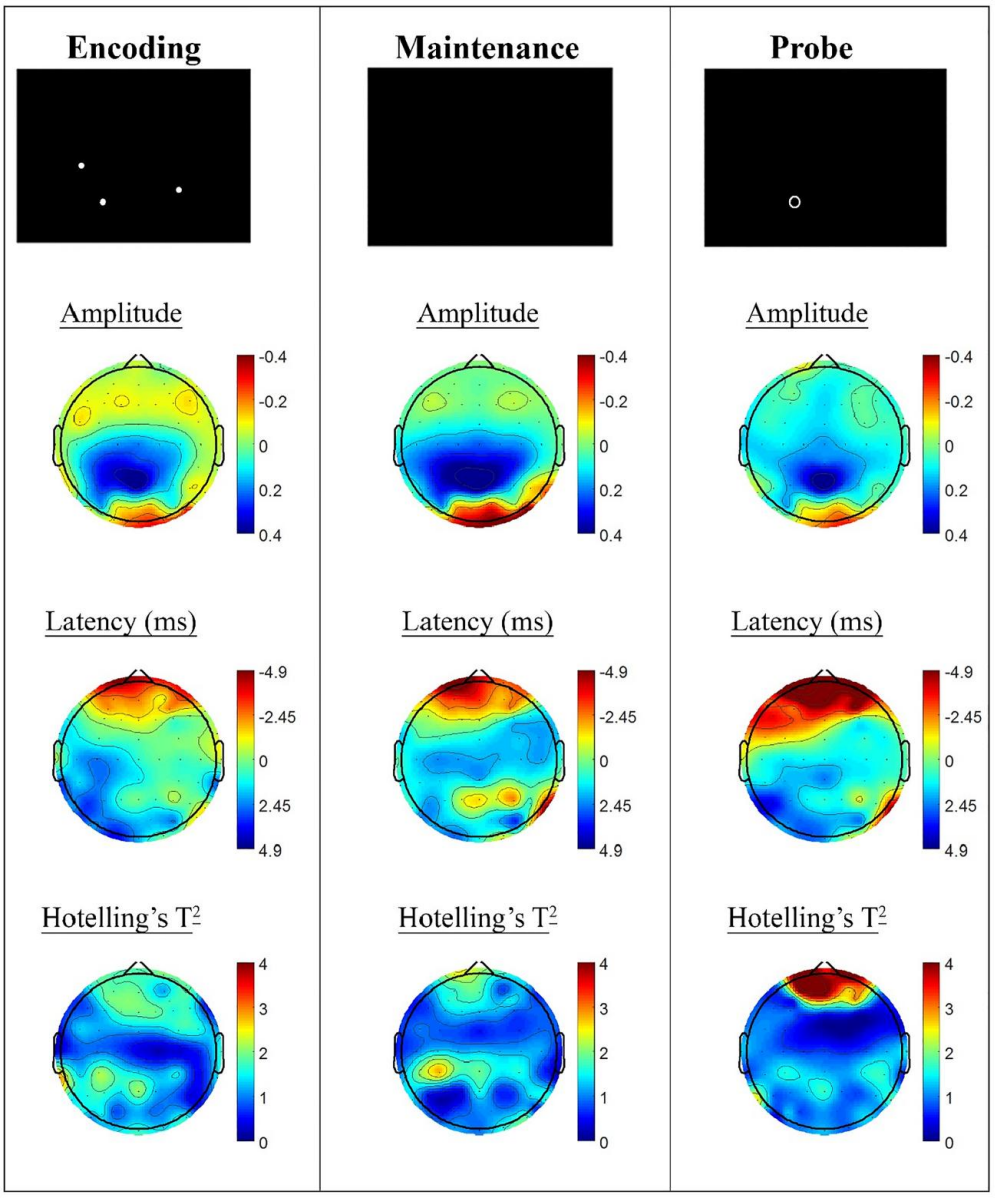
Spatial working memory (SWM) steady state visually evoked potential (SSVEP) amplitude and phase differences between *Panax quinquefolius* and placebo treatment visits, contrasting the active SWM task condition at each. Columns show topographic maps of SSVEP amplitude (top) and latency (middle), with Hotelling's T^2^ corresponding to the contrast of the two treatments for encoding (left), maintenance (middle) and retrieval (right) task segments. Hotelling's T^2^ comparisons of the two treatments showed significant latency reductions in the retrieval (probe) task phase at the *P. quinquefolius* treatment visit in prefrontal electrode sites (*p* < .01; Fp1, Fp2 and AFz; *p* < .05: Fpz and AF3)

Task windows of the active SWM task condition were contrasted between the *P. quinquefolius* treatment visit and the placebo visit, in order to quantify transient changes in SSVEP response during task processing associated with the acute dose of *P. quinquefolius*. The overall trend in these comparisons was for latency reductions in prefrontal sites, while amplitude tended to be lower in occipital sites and higher in centro‐parietal regions (see Figure 2). Hotelling's T^2^ comparisons of the two treatments showed significant latency reductions in the retrieval (probe) task phase at the *P. quinquefolius* treatment visit in prefrontal electrode sites (*p* < .01; Fp1, Fp2 and AFz; *p* < .05: Fpz and AF3). In the encoding and maintenance task segments, these trends did not reach criteria for statistical significance. No significant correlations were apparent between significant SSVEP latency changes in these electrode sites and the change in performance between treatment visits (*r*_*s*_ = .147, *p* = .651).

## 4 DISCUSSION

Twenty participants, aged between 40 and 60 years, had SSVEP recordings during completion of two cognitive tasks as part of a larger study into the cognitive effects of *P. quinquefolius*. Outcomes of the overall study have been reported elsewhere (Ossoukhova et al., 2015), in which significantly improved working memory performance was observed following *P. quinquefolius* administration. Here, we report treatment effects on SSVEP responses recorded during CP and SWM tasks completed more than 6 h post‐dose, with during recordings. Both tasks produced characteristic patterns of SSVEP response consistent with previous studies utilizing these methods to study ongoing brain activity during CP and SWM, respectively.

During SWM performance, *P. quinquefolius* was associated with significant SSVEP latency reductions within prefrontal electrodes during the retrieval component of the task. This task period requires participants to indicate whether a probe stimulus matches the location of a previously learned configuration of stimuli. Reduced SSVEP latency is typically interpreted as a manifestation of increased excitatory processes, as the phase of SSVEP response is thought to be linked with cortico‐cortical loop transmission time. As such, prefrontal latency reductions during this task component may reflect additional activation of these prefrontal regions during execution of this task, similar to the compensatory recruitment of additional prefrontal resources observed in older adults during this task (Macpherson et al., 2014). Analysis of differences in SSVEP response across treatments for the continuous performance task did not reveal any differences exceeding statistical thresholds. Furthermore, a follow‐up analysis of both tasks exploring more tonic shifts in SSVEP response, potentially linked to static shifts in inhibitory and excitatory processes, did not demonstrate any significant difference across the treatment visits.

These working memory effects on SSVEP responses occurred in the absence of any treatment‐related behavioural effects. It should be noted that in this case that SWM acts as an activation task, that is, it is designed to reliably elicit a specific pattern of activation. The task is primarily operationalised to engage these central working memory processes in a robust way and, as such, may not be optimally sensitive to detecting behavioural changes. Specifically, the quality and magnitude of the activation pattern, rather than the behavioural outcomes, may be differentially susceptible to change. This is consistent with previous findings using the same task following 30‐day administration of cocoa flavanols in a similar population (Camfield et al., 2012) and a 28‐day multivitamin intervention in a healthy young adult cohort (White et al., 2016).

Taken together with the outcomes of the broader trial (Ossoukhova et al., 2015), in which a composite working memory performance score was significantly improved, these findings provide preliminary evidence of increased recruitment of prefrontal brain regions during working memory processing, as evidenced by the SSVEP changes. This recruitment of prefrontal regions may form part of the mechanism supporting potential improvements to working memory following a single acute dose of *P. quinquefolius* Cereboost^TM^.

## CONFLICT OF INTEREST

The authors have declared no conflict of interest.

## Supporting information

Supplementary Material
