# Supplementary material for: Effects of Panax quinquefolius (American ginseng) on the steady state visually evoked potential during cognitive performance
Source: Hum Psychopharmacol. 2020 Sep 8;35(6):e2756. doi: 10.1002/hup.2756 (PMC7685123; doi:10.1002/hup.2756)
Supplement: Supplementary file 1 — Supplementary Material [file HUP-35-e2756-s001.docx]

**Supporting Information:
Effects of *Panax quinquefolius* on the steady state visually evoked potential during cognitive performance**

### SI-1: *P. quinquefolius* treatment

Participants were administered 200mg of *P. quinquefolius* Cereboost^TM^. This extract contains a standardised level of 10-12% ginsenosides and was administered in the form of an opaque capsule with maltodextrose excipient. The composition of this extract according to specific ginsenoside constituents has been described elsewhere (Scholey et al., 2010). The placebo consisted of an inert plant cellulose fibre (Avicel) that was encapsulated to be identical in appearance to the treatment. Encapsulation was performed locally by Thompsons Amcal Pharmacy, Melbourne. The treatments were prepared by a disinterested third party who took no further part in the study. Treatment order was determined by random allocation to a Latin square to ensure a fully counterbalanced design.

### SI-2: Cognitive Task Details

***A-X Continuous Performance Task***

The details of the A-X Continuous Performance Task (AX-CPT) used in the present study are identical to that described in the previous investigations of micronutrient administration (White et al., 2017; White et al., 2016). The task presents single letters at a rate of approximately 34 per minute, and involves two variants: one active task in which participants respond to a target sequence of ‘X’ preceded by ‘A’ amongst a pseudo-random sequence of letters, and a control task in which participants respond to a target letter ‘E’ amongst a predictable repeated sequence of letters (‘A’ through ‘E’). For both variants 200 stimuli, including 40 target responses, were presented across two recording runs of approximately three minutes each separated by a short break.

***Spatial Working Memory***

The spatial working memory (SWM) task adopted for this study was based on a delayed match-to-sample paradigm which has been used in studying the functional imaging correlates of spatial working memory (Jonides et al., 1993), and subsequently applied to SSVEP studies exploring age-related changes in SSVEP response (H. N. Macpherson et al., 2014), in addition to changes associated with micronutrient supplementation (H. Macpherson, Silberstein, & Pipingas, 2012; White et al., 2016). Trials presented either two or three white dots on a black background for 500 ms (encoding period), followed by presentation of a fixation cross for 3000 ms (maintenance period), after which an empty circle appeared for 1800 ms (probe). During the probe period, participants indicated whether the location of the circle enclosed an encoded location of the dot stimuli (right button press = match; left button press = new location). The control variant of the task matched sensorimotor demands of the task, however the dot stimuli remained on the screen throughout each trial. For this condition, the probe period required a response as to whether the circle stimuli either surrounded a dot (right button) or did not enclose a dot (left button). Task variants were completed in separate recording runs, both comprising 40 trials separated by a 1000 ms fixation period, half of which presented two dots during encoding and half three dots. Two runs of the active task variant were completed, resulting in 80 trials for the active task per visit and a single run of 40 trials for the control variant (to accommodate analysis of correct trials with sufficient trial numbers).

### SI-3: SSVEP recordings

***Acquisition***

Recordings were acquired from 60 scalp electrodes using a Quick-Caps electrode cap and Synamps^2^ amplifiers with Scan 4.3 Software (Neuroscan, Inc., Abbotsford, Victoria, Australia). An electrode positioned between Cz and Cpz was the on-line reference, the ground electrode was positioned between Fz and Fpz. Recordings were also obtained from left and right mastoid electrodes and data was subsequently re-referenced to linked mastoids. Interpolation was used to produce the 64 channel electrode montage described in Silberstein et al. (1990). A diffuse 13 Hz sinusoidal flicker was presented through goggles using LED arrays, superimposed over the visual field with half-silvered mirrors subtending a horizontal angle of 160° and vertical angle of 90°.

***SSVEP Processing***

All aspects of the SSVEP signal processing followed that described in recent published investigations of the neurocognitive effects of nutritional interventions (White et al., 2017; White et al., 2016), with the 13 Hz SSVEP signal extracted from ongoing recordings using established routines using in-house software (BrainSci; Silberstein, 1995b), while statistical analysis and mapping using used custom MATLAB scripts (The Mathworks Inc., Natick, MA, USA) and the EEGLAB toolbox (Delorme & Makeig, 2004). For each task the SSVEP was extracted from the raw EEG by calculating Fourier coefficients over a 20 stimulus cycle evaluation period at each electrode site, using a cosine window centered at the 13Hz stimulus frequency. This process was repeated for each subsequent window shifting one stimulus cycle for the entire duration of EEG data for each task, for each of the 64 electrodes. As part of SSVEP signal extraction, electrodes identified as having excessive artifact were replaced with the weighted average of surrounding electrodes. Excessive artifact was defined by a circular statistic exceeding 0.2 (described in Silberstein, 1995a).

In order to control for inter-individual differences in SSVEP amplitude and phase responses, normalisation of the active task variants of both AX-CPT and SWM task paradigms was conducted, using the corresponding reference task variant. Following previous acute intervention studies (White et al., 2017), to first investigate phasic task-related fluctuations in SSVEP response, this normalisation was conducted using the SSVEP response to the reference task at the same treatment visit.

***Task Blocks***

For both cognitive tasks, the SSVEP response was averaged across the three major task windows. For the A-X CPT task, mean SSVEP amplitude and phase were calculated in three windows surrounding target stimulus presentation: for the 250 ms period following the cue stimulus (‘A’; Cue), the 1500 ms hold period following (Hold), and the 1000 ms from target appearance (‘X’; Target). For the SWM task, mean SSVEP amplitude and phase were calculated for the 500 ms encoding period (Encoding), the 3000 ms maintenance period (Maintenance), and the 1000 ms from presentation of the probe stimulus (Probe).

***Statistical analysis of SSVEP responses***

Analysis of SSVEP differences were conducted with Hotelling’s T^2^, the bivariate analog to a paired *T*-test. Hotelling’s T^2^ tests for differences in the mean vector comprising the complex numbers representing the SSVEP amplitude and phase. Adjustment for multiple comparisons followed previous research to use this SSVEP method, in setting the alpha level for SSVEP analysis to 1% (adjusted *p* = .05 / 5), based on spatial principal component analysis of SSVEP data which indicated five independent components can account for over 95% of spatial variance (Silberstein & Cadusch, 1992).

Statistical comparisons assessed three separate research questions concerning the SSVEP response during performance of both AX-CPT and SWM tasks. Components of the active task were contrasted between the *P. quinquefolius* treatment visit and the placebo visit, in order to quantify transient changes in SSVEP response during task processing associated with the acute dose of *P. quinquefolius*. Supporting contrasts were calculated between active and reference tasks at the placebo visit in order to characterize the SSVEP activity associated with task performance in the absence of treatment effects (See SI-5 below). Exploratory analyses to investigate a more tonic shift in SSVEP response associated with treatment was conducted (See SI-6 below).

In the event of significant differences between the two treatment visits, the association between these SSVEP changes and behavioral performance changes from placebo to active treatment were also explored through correlations. To do so, the difference in SSVEP response at the two treatment visits was correlated with the change in task performance between these assessment visits. These correlations assessed any behavioral correlates of the SSVEP changes.

### SI-4: Analysis populations

Three criteria were used for exclusion from analysis for the two cognitive tasks: incomplete cases, excessive artifact (as defined below) or task performance approximating chance levels (<55% accuracy). Whilst single electrodes identified as having excessive artifact, as defined by a circular statistic (described in Silberstein, 1995a) exceeding 0.2, were replaced in the signal extraction process with the weighted average of surrounding electrodes, cases in which 20 or more electrodes exceeded this threshold for a given task variant, indicative of poor overall signal quality, were criteria for exclusion from analysis.

For the A-X CPT, two participants were missing SSVEP data due to technical errors (missing timing information of the SSVEP stimulus), three participants exceeded SSVEP artifact criteria, and the remaining all performed near ceiling (Mean Accuracy = 96% for both visits). As such, the analysis population for the A-X CPT was n=15 (7 completing treatment sequence A-B, and 8 completing treatment sequence B-A).

For the SWM task, all data was present, however, four participants exceeded SSVEP artifact criteria and a further four were excluded for performance approximating chance levels. The analysis population for the SWM task was n=12 (balanced for treatment order). The final analysis population for each task is summarised in Supporting Information Figure 1 below.


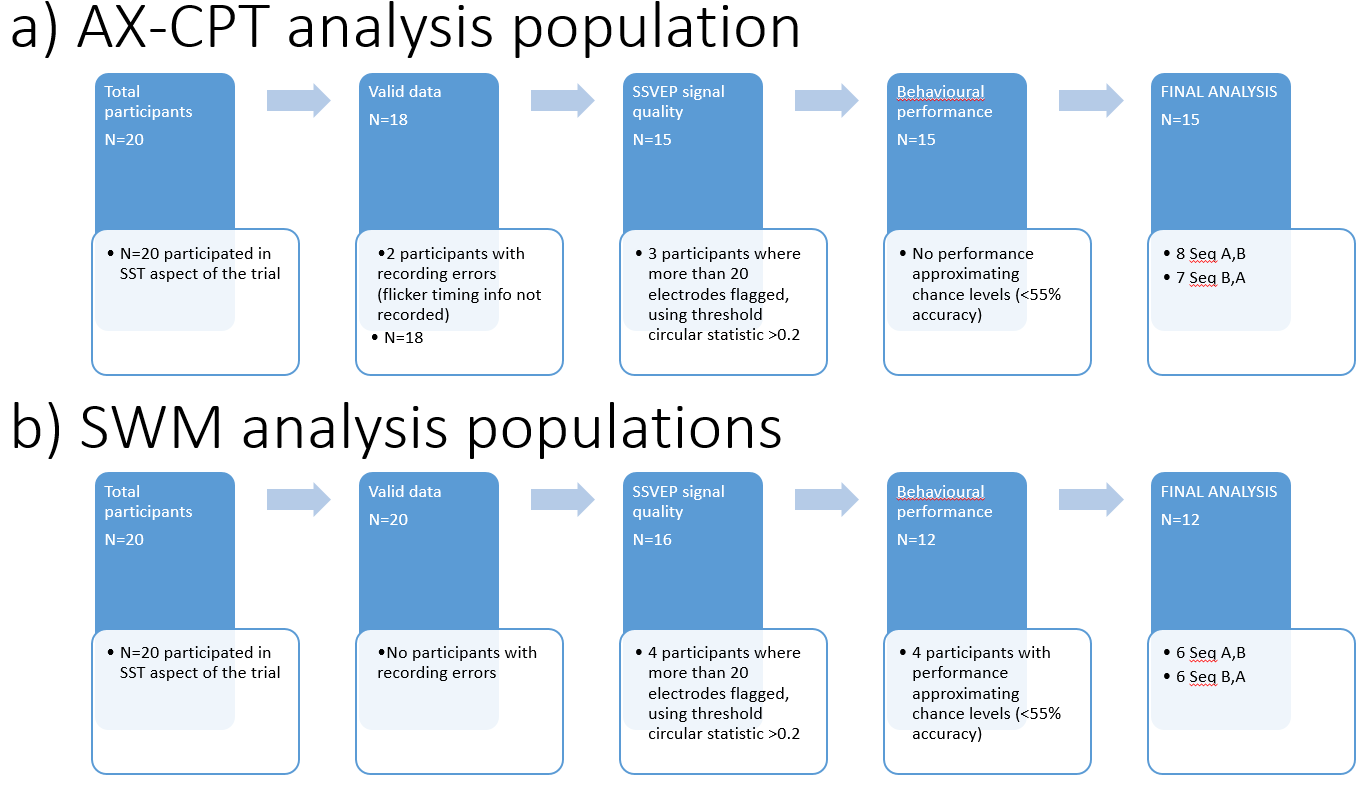


**Supporting Information Figure 1.** Process of determining final analysis populations across the two cognitive tasks.

### SI-5: SSVEP responses for each task

Contrasts were calculated between active and reference tasks at the placebo visit in order to characterize the SSVEP activity associated with task performance in the absence of treatment effects.

***AX-CPT performance***

Contrasts were calculated between the active (A-X variant) and reference (ABCDE) continuous performance tasks at the placebo visit in order to characterize the SSVEP activity associated with task performance. Supporting Information Figure 2 shows a pattern of reduced amplitude and latency across frontal electrode sites across the three task phases. The statistical assessment of these differences only exceeded criteria for significance in a single right lateralized parieto-temporal electrode during Cue stimulus presentation, which also showed this reduced amplitude and latency in the active task condition. Across the prefrontal and posterior regions showing reduced amplitude and latency, results did not reach criteria for significance after adjusting for multiple comparisons (uncorrected p<.05; Cue period: two prefrontal electrodes and one occipital electrode; Hold period: six frontal electrodes; Target period: two frontal electrodes). While these patterns must be interpreted with caution given the statistical strength of the findings, the observed amplitude and latency reductions are consistent with previous studies to explore SSVEP responses to A-X CPT performance (Silberstein et al., 1998; Silberstein, Line, Pipingas, Copolov, & Harris, 2000; White et al., 2017; White et al., 2016), further justifying the use of the task as a well-characterised paradigm for studying treatment related fluctuations in ongoing activity.


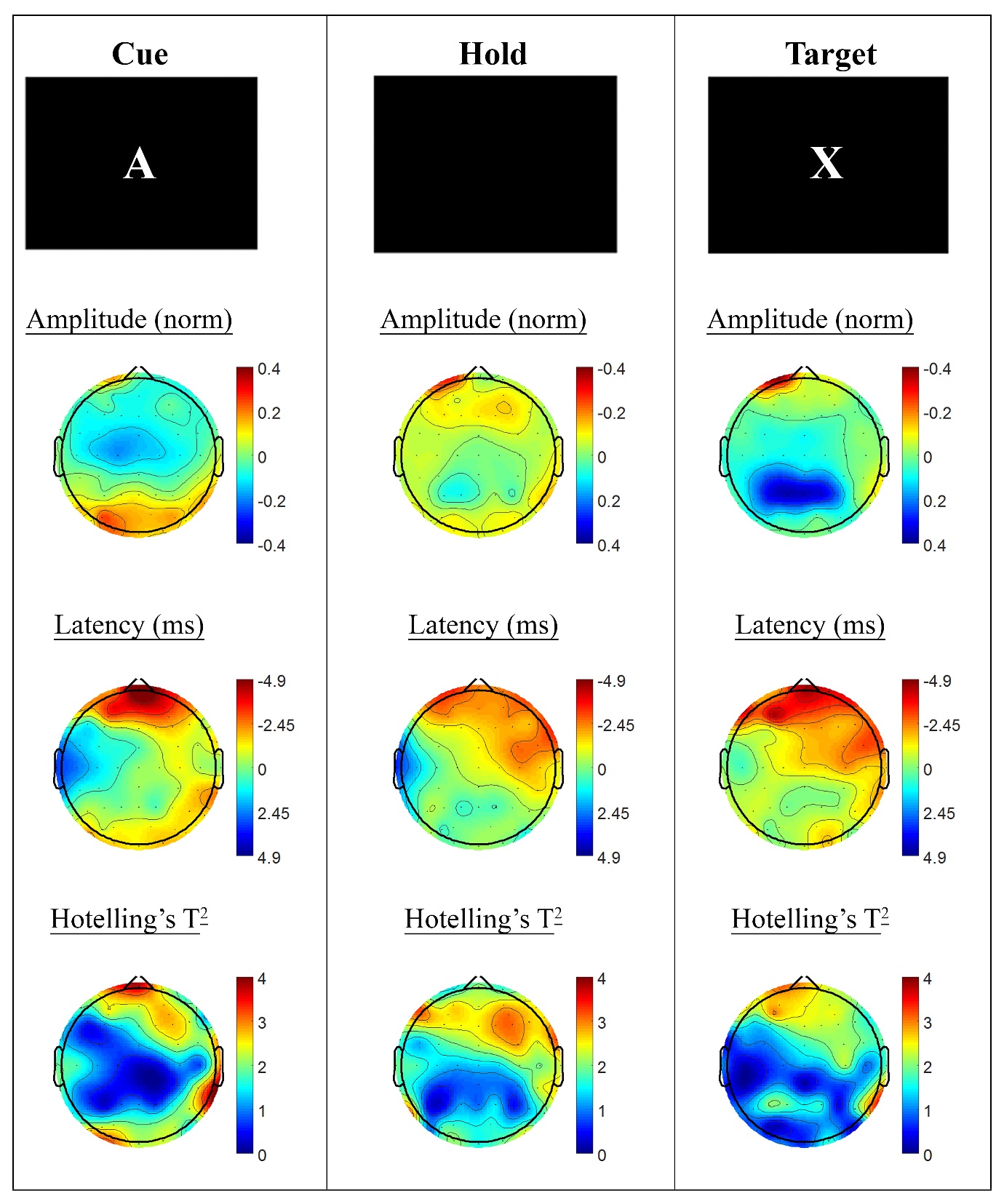


**Supporting Information Figure 2.** A-X CPT SSVEP at the placebo treatment visit. SSVEP amplitude and phase differences, with respect to the control task. Columns show the three task blocks, with topographic maps of SSVEP amplitude (top) and phase (as latency, middle) and the Hotelling’s T2 (bottom) corresponding to the contrast of active and control task variants for Cue (left), Hold (middle) and Target (right) task segments.

***SWM performance***

In order to characterize the SSVEP activity associated with task performance in the absence of treatment effects, the active and reference variants of the SWM task were first contrasted at the placebo visit. Supporting Information Figure 3 shows the SSVEP response to SWM task completion. Significant differences were observed between active and reference task variants at the placebo visit in the Retrieval (probe) task blocks, where significantly increased SSVEP latency was observed in left temporo-parietal electrodes (*p*<.01; TP7, CP5). Similar patterns were also observed in right temporo-parietal regions in both the Maintenance and Retrieval blocks (*p*<.05, uncorrected; Maintenance: TP8, T6; Retrieval: CP6, TP8, T6). Further patterns of activity, whilst not statistically significant, were consistent with previous research, showing frontal latency reductions during encoding and increased amplitude during the maintenance period.


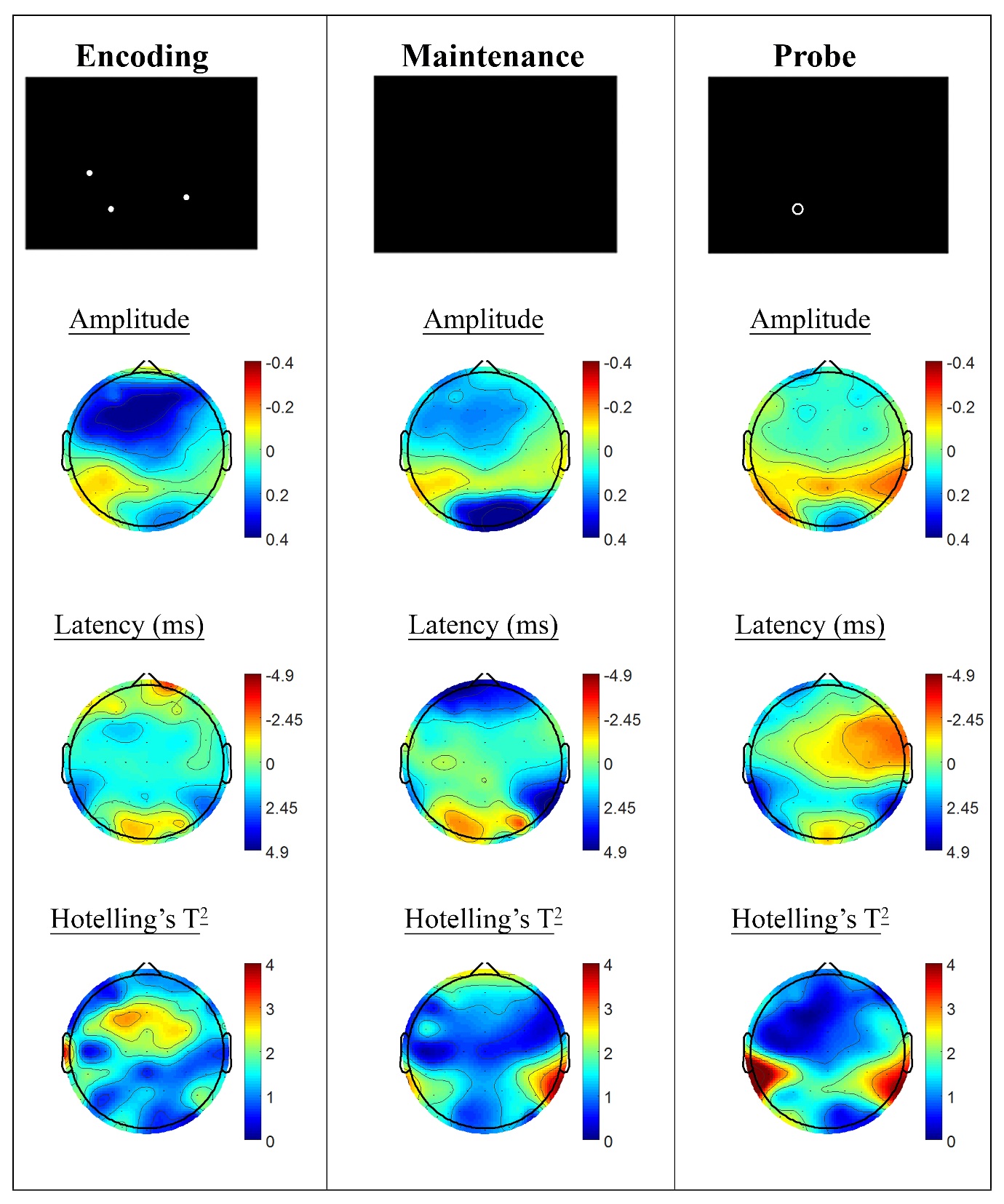


**Supporting Information Figure 3.** Spatial Working Memory SSVEP amplitude and phase differences, contrasted with the control task, at the placebo assessment across the sample. Columns show topographic maps of SSVEP amplitude (top) and latency (middle), with Hotelling’s T2 corresponding to the contrast of active and control task variants for Encoding (left), Maintenance (middle) and Retrieval (right) task segments.

### SI-6: Tonic SSVEP responses

Tonic changes in SSVEP response were probed by collapsing the three major task components to a single averaging window. This analysis utilised the reference task at the placebo visit to normalise data from both treatment visits to allow further exploration of any tonic shifts in SSVEP response associated with *P. quinquefolius* administration, as all SSVEP responses were relative to mean SSVEP response during performance of reference tasks at the placebo visit. This approach has previously proven sensitive to generalised shifts towards excitatory processes in acute interventions containing caffeine (White et al., 2017).

No significant differences were observed for this analysis (with a single temporal electrode uncorrected p<.05 for the AX-CPT, in which increased latency was observed at the active treatment visit). The results of these analyses are shown in Supporting Information Figure 4 below.


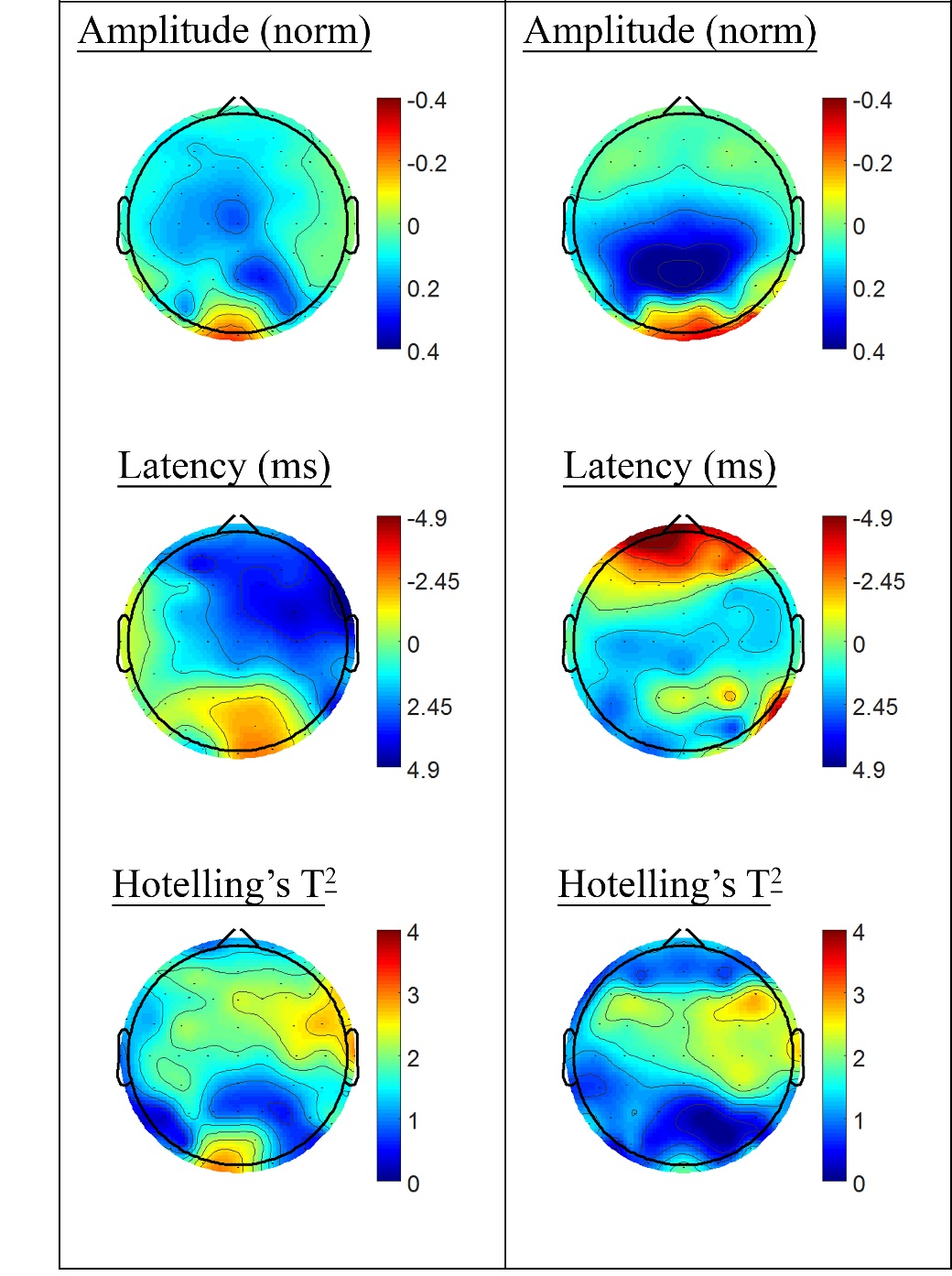


**Supporting Information Figure 4.** Tonic changes in SSVEP response associated with treatment, averaged across a large window of task performance for both AX-CPT (left) and SWM (right) tasks. Topographic maps (from top) show amplitude and phase (as latency in ms) differences between treatment and placebo, with the bottom plot showing Hotelling’s T2 values plotted for corresponding contrast of treatment visits. Warm colours indicate amplitude and latency reductions and lower P-values respectively.

### Supporting Information References:

Delorme, A., & Makeig, S. (2004). EEGLAB: an open source toolbox for analysis of single-trial EEG dynamics including independent component analysis. *J Neurosci Methods, 134*(1), 9-21. doi:10.1016/j.jneumeth.2003.10.009

Jonides, J., Smith, E. E., Koeppe, R. A., Awh, E., Minoshima, S., & Mintun, M. A. (1993). Spatial working memory in humans as revealed by PET. *Nature, 363*(6430), 623-625. doi:10.1038/363623a0

Macpherson, H., Silberstein, R., & Pipingas, A. (2012). Neurocognitive effects of multivitamin supplementation on the steady state visually evoked potential (SSVEP) measure of brain activity in elderly women. *Physiol Behav, 107*(3), 346-354. doi:10.1016/j.physbeh.2012.08.006

Macpherson, H. N., White, D. J., Ellis, K. A., Stough, C., Camfield, D., Silberstein, R., & Pipingas, A. (2014). Age-related changes to the neural correlates of working memory which emerge after midlife. *Front. Aging Neurosci., 6*, 70. doi:10.3389/fnagi.2014.00070

Scholey, A., Ossoukhova, A., Owen, L., Ibarra, A., Pipingas, A., He, K., . . . Stough, C. (2010). Effects of American ginseng (Panax quinquefolius) on neurocognitive function: An acute, randomised, double-blind, placebo-controlled, crossover study. *Psychopharmacology, 212*(3), 345-356. doi:10.1007/s00213-010-1964-y

Silberstein, R. B. (1995a). Neuromodulation of neocortical dynamics. In P. Nunez (Ed.), *Neocortical Dynamics and Human EEG Rhythms* (pp. 591-627). New York: Oxford University Press.

Silberstein, R. B. (1995b). Steady-state visually evoked potentials, brain resonances, and cognitive processes. In P. Nunez (Ed.), *Neocortical Dynamics and Human EEG Rhythms* (pp. 272-303). New York: Oxford University Press.

Silberstein, R. B., & Cadusch, P. J. (1992). Measurement processes and spatial principal components analysis. *Brain Topogr, 4*(4), 267-276.

Silberstein, R. B., Farrow, M., Levy, F., Pipingas, A., Hay, D. A., & Jarman, F. C. (1998). Functional brain electrical activity mapping in boys with attention-deficit/hyperactivity disorder. *Arch Gen Psychiatry, 55*(12), 1105-1112. doi:10.1001/archpsyc.55.12.1105

Silberstein, R. B., Line, P., Pipingas, A., Copolov, D., & Harris, P. (2000). Steady-state visually evoked potential topography during the continuous performance task in normal controls and schizophrenia. *Clin Neurophysiol, 111*(5), 850-857. doi:10.1016/S1388-2457(99)00324-7

Silberstein, R. B., Schier, M. A., Pipingas, A., Ciorciari, J., Wood, S. R., & Simpson, D. G. (1990). Steady-state Visually Evoked Potential Topography associated with a visual vigilance task. *Brain Topography, 3*(2), 337-347.

White, D. J., Camfield, D. A., Maggini, S., Pipingas, A., Silberstein, R., Stough, C., & Scholey, A. (2017). The effect of a single dose of multivitamin and mineral combinations with and without guarana on functional brain activity during a continuous performance task. *Nutr Neurosci, 20*(1), 8-22. doi:10.1179/1476830514Y.0000000157

White, D. J., Cox, K. H., Hughes, M. E., Pipingas, A., Peters, R., & Scholey, A. B. (2016). Functional Brain Activity Changes after 4 Weeks Supplementation with a Multi-Vitamin/Mineral Combination: A Randomized, Double-Blind, Placebo-Controlled Trial Exploring Functional Magnetic Resonance Imaging and Steady-State Visual Evoked Potentials during Working Memory. *Front Aging Neurosci, 8*(288), 288. doi:10.3389/fnagi.2016.00288
